# Efficacy of Combined Formulations of Fungicides with Different Modes of Action in Controlling Botrytis Gray Mold Disease in Chickpea

**DOI:** 10.1155/2014/639246

**Published:** 2014-03-02

**Authors:** M. H. Rashid, M. Ashraf Hossain, M. A. Kashem, Shiv Kumar, M. Y. Rafii, M. A. Latif

**Affiliations:** ^1^RARS, Bangladesh Agricultural Research Institute, Rahmatpur, Barisal, Bangladesh; ^2^Pulses Research Centre, BARI, Gazipur, Bangladesh; ^3^Bangladesh Institute of Nuclear Agriculture, Mymensingh, Bangladesh; ^4^International Centre for Agricultural Research in the Dry Areas, Aleppo, Syria; ^5^Institute of Tropical Agriculture, Universiti Putra Malaysia, 43400 Serdang, Selangor, Malaysia; ^6^Department of Crop Science, Faculty of Agriculture, Universiti Putra Malaysia, 43400 Serdang, Selangor, Malaysia; ^7^Plant Pathology Division, Bangladesh Rice Research Institute, Gazipur 1701, Bangladesh

## Abstract

Botrytis gray mold (BGM) caused by *Botrytis cinerea* Pers. Ex. Fr. is an extremely devastating disease of chickpea (*Cicer arietinum* L.) and has a regional as well as an international perspective. Unfortunately, nonchemical methods for its control are weak and ineffective. In order to identify an effective control measure, six fungicides with different modes of action were evaluated on a BGM susceptible chickpea variety BARIchhola-1 at a high BGM incidence location (Madaripur) in Bangladesh for three years (2008, 2009, and 2010). Among the six fungicides tested, one was protectant [Vondozeb 42SC, a.i. mancozeb (0.2%)], two systemic [Bavistin 50 WP, a.i. carbendazim (0.2%), and Protaf 250EC, propiconazole (0.05%)], and three combination formulations [Acrobat MZ690, dimethomorph 9% + mancozeb 60%, (0.2%); Secure 600 WG, phenomadone + mancozeb (0.2%); and Companion, mancozeb 63% + carbendazim 12% (0.2%)]. The results showed superiority of combination formulations involving both protectant and systemic fungicides over the sole application of either fungicide separately. Among the combination fungicides, Companion was most effective, resulting in the lowest disease severity (3.33 score on 1–9 scale) and the highest increase (38%) of grain yield in chickpea. Therefore, this product could be preferred over the sole application of either solo protectant or systemic fungicides to reduce yield losses and avoid fungicide resistance.

## 1. Introduction

Globally, chickpea (*Cicer arietinum *L.) is the third most important pulse crop after dry beans and dry peas and is presently grown on 11.99 million ha with 10.94 million tons production [1]. Among the various biotic stresses of chickpea, botrytis gray mold (BGM) caused by *Botrytis cinerea* Pers. Ex. Fr. is the most devastating disease and has both regional and international perspective. It is an economically important disease in areas with cool, cloudy, and humid weather [2]. The crop encounters frequent BGM epidemics with near complete yield loss in the Indian Subcontinent accounting for 80% of the global chickpea area. Frequent BGM epidemics in north Indian states have been one of the reasons for a geographical shift of chickpea cultivation to southern states [3]. In Nepal, the recurring problem of BGM has caused a drastic reduction in chickpea area from 28,190 ha in 1990 to less than 9,000 ha in 2010. In Bangladesh, a conservative estimate of crop loss due to BGM ranges from 10 to 15% under normal conditions but can reach up to 80% or more under periods of high disease pressure [4, 5]. Therefore, chickpea cultivation has declined sharply from 102,867 ha in 1990 to 18,219 ha in 2000 [6] and further reduced to 7,224 ha in recent years. Yield losses due to BGM have also been reported from other chickpea growing countries including Australia, Argentina, Canada, Columbia, Hungary, Mexico, Myanmar, Spain, Turkey, the USA, and Vietnam [2, 7].

The fungus *Botrytis cinerea *is an opportunistic pathogen on a wide variety of crops, causing gray mold disease primarily through infections via wounds or dead plant parts. Various synthetic fungicides for controlling this disease have become ineffective due to the development of resistance [8, 9]. In chickpea, BGM is difficult to manage as the causal pathogen is soil, seed, and air borne and, unfortunately, nonchemical methods for its control are difficult and ineffective. Extensive screening of chickpea germplasm against BGM did not identify a genotype with a high level of resistance [2, 6, 10–16]. Therefore, integration of chemical application with cultural practices such as late sowing of erect cultivars at lower plant densities remains the only option to manage the disease commercially [5]. For chemical control, numerous fungicides with different modes of action are commercially available. Seed treatments with iprodione, mancozeb, thiabendazole, triadimefon, triadimenol, vinclozolin, thiram, benomyl, carbendazim, or cantan are effective in reducing seed infection [2, 5, 17–24]. Foliar sprays with captan, carbendazim, chlorothalonil, mancozeb, thiabendazole, thiram, triadimefon, or vinecloolin can offer some level of moderate control particularly when used in combination with seed dressing fungicides [5, 18]. All these fungicides belong to either protectant or systemic group of chemicals.

Traditionally, seed treatments with protectant fungicides have been utilized for controlling BGM of chickpea in Bangladesh. Protectant fungicides do not offer durable control against the disease since they are easily washed off the crop after rainfall. In Bangladesh, when rainfall is frequent, systemic fungicides often perform better than the protectant fungicides. It is reported that intense use of systemic fungicides especially benzimidazoles selects for resistant fungal strains of *Botrytis cinerea* across the crops [9, 25–27]. It is therefore recommended to use systemic fungicides responsibly for the control of BGM in chickpea. The use of formulated mixtures (coformulations) of two fungicides with different modes of action has often been recommended to help manage fungicide resistance development [28–30]. Recently, fungicides combining both protectant and systemic chemicals have been formulated and made commercially available to farmers mainly broaden the spectrum of activity to delay selection of resistant fungal populations and 8 to optimize efficacy [31]. The present study was, therefore, undertaken to assess the efficacy of coformulations in comparison to solo applications of protectant or systemic fungicides separately in controlling BGM disease in chickpea.

## 2. Materials and Methods 

Field experiments were conducted at the Pulse Research Substation of Bangladesh Agricultural Research Institute (Madaripur) Bangladesh in the postrainy season of three consecutive years (2007-08, 2008-09, and 2009-10). The experimental site is a known high incidence area for BGM disease in Bangladesh due to the environmental conditions prevalent during the crop season such as high relative humidity (>70%) and optimum air temperature (20–28°C) [4]. The experiment design was a randomized complete block with four replications. A BGM susceptible variety BARIchhola-1 was planted in rows spaced 40 cm apart in 12 m^2^ (4 m × 3 m) plots. Six commercially available fungicides representing different modes of action, including systemic [Bavistin 50WP (carbendazim), BASF Bangladesh Ltd., and Protaf 250 EC (propiconazole), Auto Crop Care Ltd.)], protectant [Vondozeb 42 SC (mancozeb), Naafco Pvt. Ltd.], and combination of formulations [Acrobat MZ690 (dimethomorph 9% + mancozeb 60%), BASF Bangladesh Ltd.; Secure 600WG (phenomadone + mancozeb), Bayer CropScience Ltd.; and Companion (mancozeb 63% + carbendazim 12%), Auto Crop Care Ltd.], and combination formulations [Acrobat MZ690 (dimethomorph 9% + mancozeb 60%), BASF Bangladesh Ltd.; Secure 600 WG (phenomadone + mancozeb), Bayer CropScience Ltd.; and Companion (mancozeb 63% + carbendazim 12%), Auto Crop Care Ltd.] were tested along with a nontreated control plot. These fungicides were applied as a foliar spray before the onset of the disease @ 0.2% except for Protaf 250 EC at 0.5%. First spray was done using a backpack low volume sprayer equipped with cone nozzle just before the onset of flowering stage followed by three subsequent sprays at 10-day intervals. Standard cultural practices typical to the area were achieved for weeding, insecticide, and fertility management. Insecticide applications were done as necessary. Insecticide Karate (0.2%) was applied to protect the crop from the infestation of *Helicoverpa* pod borer.

BGM occurred naturally and assessments were made three times: at 50% flowering stage, 10 days thereafter, and at 100% flowering stage. Disease assessment was based on a 1–9 scale [2]. In addition, assessments of plant height (cm), number of pods/plant, number of seeds/pod, and 100-seed weight (g) were recorded on 10 randomly selected plants from each plot. Grain yield (kg/ha) was estimated based on the yield harvested from the whole plot.

Data were evaluated using ANOVA and analyzed by MSTAT software with fungicide treatment in different years as fixed effects and blocks × blocks (year) as random effects. The nontreated control plot was used for comparing the relative performance of the treatments. Treatment means were separated by Fisher's Protected LSD at 0.05 probability level.

## 3. Results and Discussion

ANOVA showed highly significant differences (*P* < 0.01) among the treatments for disease severity, grain yield, and number of pods/plant in individual years. Pooled analysis showed highly significant differences among the fungicide treatments over the years. Nonsignificant effects of year and treatment × year interaction suggested that the efficacy of fungicide treatments was superior to nontreated control plots across the years for disease severity, grain yield, and number of pods/plant. Therefore, data were summarized over the three-year period for comparison. For plant height, number of seeds/pod, and 100-seed weight, fungicide treatments were superior to nontreated control plots numerically but not statistically.

### 3.1. BGM Disease Intensity

**Figure 1:**
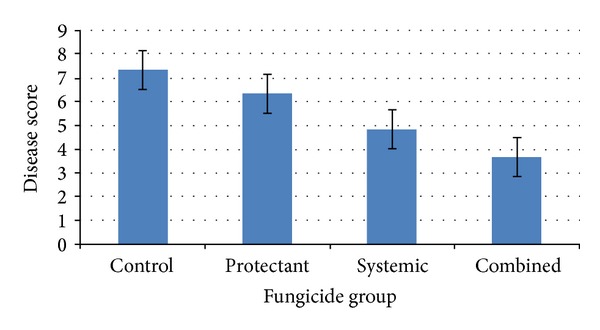
Effect of fungicides with different modes of action on Botrytis gray mold disease severity in susceptible chickpea variety “BARIchhola-1.”

Results of foliar spray of the fungicides on BGM in chickpea variety BARIchhola-1 showed significant reduction in disease severity with all the treatments compared to nontreated control plot (Table 1). The results on disease severity were similar over three years of experimentation and, therefore, data were averaged over the years for comparison. Coformulations involving both protectant and systemic fungicides showed superiority over the sole application of either systemic or protectant fungicides and, in turn, systemic fungicides were superior to protectant fungicides in controlling the disease (Figure 1). The average disease index was 3.67 with coformulations followed by 4.84 with systemic and 6.33 with protectant fungicides, compared to 7.33 in nontreated control plot. It is reported that while the protectant fungicides prevent only spore germination, the systemic fungicides inhibit fungal growth and sporulation [32]. Coformulations have the advantage of both not when the systemic partner is not active and, thus, their superiority was probably due to reduced infection at the onset of disease at early flowering stage and later on reduced growth and sporulation of the pathogen. Among the coformulations, foliar spray of Companion kept the disease minimum at 3.3 on a 1–9 scale (Table 1). Secure 600 WG was the second most effective fungicide followed by Bavistin 50 WP and Acrobat MZ in reducing disease severity. Earlier studies also reported superior efficacy of combined formulation of carbendazim + mancozeb in controlling collar and root rot diseases of strawberry and chilli caused by *Sclerotium rolfsii* [31, 33] and BGM disease of paprika caused by *Botrytis cinerea* [34]. In the present study, Companion showed additive effect, combining the advantage of both mancozeb and carbendazim. There have been many reports on the uses of mixtures of synthetic fungicides for the control of plant pathogenic fungi. When utilized in two-way mixtures, such fungicides maintain or enhance the level of control of a pathogen at reduced rates for both components utilized in combinations, compared to solo applied at higher rates [8, 35]. Therefore, coformulations should be preferred for effective control of BGM in chickpea.

### 3.2. Grain Yield

**Figure 2:**
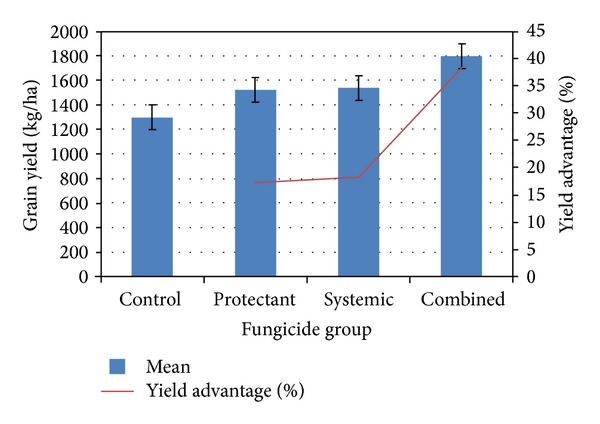
Effect of fungicides on grain yield of BGM susceptible chickpea variety “BARIchhola-1.” Values are means of four replicates over three years.

Grain yield was significantly higher in all the fungicide treatment plots than in the nontreated control plot (Table 1). Yearwise and pooled analysis showed that coformulations had a significant yield advantage over the sole application of either systemic or protectant fungicides (Figure 2). Coformulations showed 38% increase in grain yield over the nontreated control plot, 18% over protectant, and 17% over systemic fungicides. Systemic and protectant fungicides were equally effective against BGM in terms of yield (17%) over the nontreated control plot. Companion was the most effective fungicide followed by Secure 600 WG, Acrobat MZ, and Bavistin 50 WP in increasing grain yield (Table 1). The highest average yield was achieved with Companion (1921 kg/ha) followed by Secure 600 WG (1766 kg/ha), Acrobat MZ (1699 kg/ha), and Bavistin 50WP (1598 kg/ha) compared to 1298 kg/ha in nontreated control plot. ANOVA revealed highly significant negative correlation (*r* = −0.653**) between grain yield and BGM disease intensity in chickpea (Table 4), indicating that grain yield reduced sharply with increased disease intensity and fungicides are useful to harvest higher chickpea yield in BGM prone areas.

### 3.3. Yield Components

The results presented in Tables 2 and 3 showed that combination formulations were superior to sole application of the fungicide components individually in terms of various yield components. However, their effect was statistically significant only for number of pods per plant and only numerically superior for number of seeds per pod, plant height, and 100-seed weight. The average number of pods per plant was highest in the Companion treated plots and lowest in the nontreated control plot. The average values for plant height, seeds/pod, and 100-seed weight were highest with foliar spray of Companion and lowest in the nontreated control plot. These results confirmed the earlier findings of [18, 36] that seed treatments with contact fungicides coupled with foliar spray with systemic fungicides provided better control over the individual control measures against BGM in chickpea. The results showed that the disease intensity had significantly negative correlations with number of pods per plant (*r* = −0.730**) and seeds per pod (*r* = −0.533**), suggesting that BGM in chickpea reduced grain yield mainly because of reduction in pods/plant and seeds/pod. This is expected as the BGM disease initiates just before the onset of flowering, a critical stage which decides the plant capacity to bear the pods which, in turn, is highly positively correlated with grain yield (*r* = 0.630**).

## 4. Conclusion

Overall, the study clearly showed strong effect of fungicide applications on the reduction of disease severity and increase in grain yield and number of pods per plant. Coformulations showed additive effect of the protectant and systemic fungicides over solo application of each component fungicide in BGM management of chickpea. In the absence of host resistance, coformulations should be preferred over solo applications of systemic single-site fungicides to avoid resistance. Coformulations gave better disease control and higher grain yield in chickpea in BGM prone areas of South Asia than solo fungicides.

## Figures and Tables

**Table 1:** Effect of foliar spray of fungicides with different modes of action on BGM disease severity and grain yield in BGM susceptible chickpea variety “BARIchhola-1” under field conditions at Madaripur in Bangladesh during postrainy seasons of 2007-08, 2008-09, and 2009-10.

Treatments	Disease severity (1–9 score)				Grain yield (kg/ha)				Yield increase over control (%)
	2007-08	2008-09	2009-10	Mean	2007-08	2008-09	2009-10	Mean	
Systemic fungicides									
Bavistin 50 WP	4	4	4	4.00	1610	1478	1705	1598	23.11
Protaf 250 EC	6	5	6	5.67	1552	1212	1651	1472	13.40
Protectant fungicides									
Vondozeb 42 SC	6	7	6	6.33	1402	1369	1793	1521	17.18
Combination formulation									
Acrobat MZ	4	4	4	4.00	1695	1713	1688	1699	30.89
Secure 600 WG	3	4	4	3.67	1703	1686	1910	1766	36.06
Companion	3	3	4	3.33	1785	1804	2174	1921	48.00

Mean	4	5	5	5	1625	1544	1820	1663	28
Control	8	7	7	7.33	1218	1139	1536	1298	—
CV (%)	—	—	—	—	12.35	12.35	7.94	—	—
LSD (0.05)	—	—	—	—	120.6	231.26	317.4	—	—

**Table 2:** Effect of foliar spray of fungicides with different modes of action on plant height and number of pods/plant in BGM susceptible chickpea variety “BARIchhola-1” under field conditions at Madaripur in Bangladesh during postrainy season of 2007-08, 2008-09, and 2009-10.

Treatments	Plant height (cm)				Number of pods/plant			
	2007-08	2008-09	2009-10	Average	2007-08	2008-09	2009-10	Average
Bavistin 50 WP	54.00	46.77	52.50	51.09	37.70	42.17	36.73	38.87
Protaf 250 EC	52.40	45.93	51.63	49.99	36.23	34.27	33.33	34.61
Vondozeb 42 SC	55.10	46.77	56.20	52.69	35.47	46.57	38.60	40.21
Acrobat MZ	55.40	49.83	56.33	53.85	44.37	46.70	47.27	46.11
Secure 600 WG	54.80	46.20	56.43	52.48	41.70	47.17	45.40	44.76
Companion	55.60	48.60	57.60	53.93	53.57	53.57	47.47	51.54
Control	48.55	45.57	52.30	48.81	29.90	31.87	30.93	30.90

CV (%)	8.21	11.00	9.86	—	12.17	13.13	27.35	—
LSD (0.05)	7.33	9.75	9.63	—	5.48	6.16	18.35	—

**Table 3:** Effect of fungicides with modes of action on number of seeds/pod and 100-seed weight in BGM susceptible chickpea variety “BARIchhola-1” under field conditions at Madaripur in Bangladesh during the postrainy season of 2007-08, 2008-09, and 2009-10.

Treatments	Number of seeds/pod				100-seed weight (g)			
	2007-08	2008-09	2009-10	Mean	2007-08	2008-09	2009-10	Mean
Bavistin 50 WP	1.52	1.55	1.37	1.48	12.17	11.83	15.67	13.22
Protaf 250 EC	1.57	1.53	1.50	1.53	11.93	10.27	15.00	12.40
Vondozeb 42 SC	1.55	1.53	1.30	1.46	11.43	12.33	13.00	12.25
Acrobat MZ	1.59	1.59	1.50	1.56	12.73	12.13	15.03	13.30
Secure 600 WG	1.55	1.60	1.53	1.56	12.43	12.00	15.00	13.14
Companion	1.59	1.63	1.51	1.58	13.00	12.43	15.00	13.48
Control	1.50	1.50	1.27	1.42	11.33	10.93	14.33	12.20

CV (%)	8.71	8.69	10.35	—	10.11	10.11	7.33	—
LSD (0.05)	0.18	0.15	0.29	—	2.05	0.65	1.92	—

**Table 4:** Correlation coefficient values of BGM disease intensity with grain yield and yield components in BGM susceptible chickpea variety “BARIchhola-1”.

Trait	Grain yield	Pods/plant	Plant height	Seeds/pod	Seed weight
BGM score	−0.653**	−0.730**	−0.319	−0.533**	−0.236
Grain yield		0.630**	0.677**	0.062	0.681**
Pods/plant			0.198	0.524**	0.189
Plant height				−0.208	0.598**
Seeds/pod					−0.305

**Significant at 1% level; “*r*” values are based on observations recorded over three years.
